# β-adrenergic receptor inhibition enhances oncolytic herpes virus propagation through STAT3 activation in gastric cancer

**DOI:** 10.1186/s13578-021-00687-1

**Published:** 2021-09-20

**Authors:** Jiali Hu, Ruitao Lu, Yu Zhang, Wei Li, Qian Hu, Cuiyu Chen, Zhaoqian Liu, Wei Zhang, Ling Chen, Ran Xu, Jia Luo, Howard L. McLeod, Yijing He

**Affiliations:** 1 Department of Clinical Pharmacology, Xiangya Hospital, Central South University, Changsha, 410000 Hunan China; 2 Institute of Clinical Pharmacology, Hunan Key Laboratory of Pharmacogenetics, Central South University, Changsha, Hunan China; 3 School of Basic Medical Sciences, Guangzhou Medical University, Guangzhou, Guangdong China; 4 Department of Gastrointestinal Surgery, Xiangya Hospital, Central South University, Changsha, Hunan China; 5 National Clinical Research Center for Geriatric Disorders, Xiangya Hospital, Central South University, Changsha, Hunan China; 6 Department of Urology, The Second Xiangya Hospital of Central South University, Changsha, Hunan China; 7 Department of Hepatobiliary and Intestinal Surgery, Hunan Cancer Hospital, Changsha, Hunan China; 8 Zhuhai People’s Hospital (Zhuhai Hospital Affiliated With Jinan University), Zhuhai, Guangdong China; 9 Geriatric Oncology Consortium, Tampa, FL USA

**Keywords:** Propranolol, Oncolytic virus, Gastric cancer, Innate immunity, Type I IFN signaling

## Abstract

**Background:**

Oncolytic viruses (OVs) are considered a promising therapeutic alternative for cancer. However, OVs could activate the host innate immunity, then impair the viral propagation in tumor cells. In this study, we explored the effect of propranolol, a non-selective β-blocker, on the antitumor efficacy of T1012G virus in gastric cancer models.

**Methods:**

The proliferation of gastric cancer cells treated with monotherapy or combination treatment was detected by CCK8 cell proliferation assay. The effect of propranolol was further evaluated by in vitro viral replication assays. In vivo tumor xenograft experiments were used to observe the effect of combination therapy on gastric cancer growth in mice. The expression levels of viral proteins and interferon responsive genes were detected in the gastric cancer cell lines treated with combined treatment by western blot. The impact of propranolol on IFN-α/β-mediated inhibition of viral propagation and the expression of antiviral gene PKR was detected by viral replication assays and western blot.

**Results:**

Cell viability assay detected a 97.9% decrease of T1012G IC50 in HGC-27 when it was pretreated with propranolol along with a sevenfold increase of virus titers compared with T1012G only group (*P* < 0.001). Moreover, propranolol pretreatment caused sustained tumor regression (335.3 ± 36.92 mm^3^ vs. 1118 ± 210.0 mm^3^, *P* < 0.01) and enhanced the viral propagation (fourfold increase, *P* < 0.01) compared with T1012G only group. Propranolol pretreatment significantly enhanced the p-STAT3 (2.9-fold, *P* < 0.05) and suppressed p-PKR (65.94% ± 10.11%, *P* < 0.05) compared with T1012G only group. In addition, propranolol could counteract IFN-α/β-mediated inhibition of viral propagation (compared with IFNα: 5.1-fold, *P* < 0.001; IFNβ: 4.6-fold, *P* < 0.01) or enhancement of PKR activation (IFNα: 92.57% ± 1.77%, *P* < 0.001, IFNβ: 99.34% ± 0.13% decrease, *P* < 0.001).

**Conclusions:**

In summary, β-blocker pretreatment could improve the propagation and therapeutic efficacy of T1012G in human gastric cancer by regulating STAT3-PKR signaling cascade, even in the presence of type I IFNs. These data support new strategies of improving the efficacy of OVs in gastric cancer.

**Supplementary Information:**

The online version contains supplementary material available at 10.1186/s13578-021-00687-1.

## Background

Oncolytic viruses (OVs) are a group of genetically modified viruses that selectively replicate in tumor cells and induce host antitumor immunity, which mainly uses herpesvirus, adenovirus, and reovirus, etc., as backbone [1]. A large number of preclinical studies have confirmed that OVs could effectively control tumor growth through direct oncolytic killing effect and enhancing anti-tumor immune response, including breast cancer, colorectal cancer, gastric cancer, melanoma, prostate cancer, etc. [2–4]. Talimogene laherparepvec (T-VEC) is a genetically modified Type I herpes simplex virus that was the first OVs showed clinical benefit in patients with melanoma [5]. With the approval of T-VEC by the US FDA in 2015 [6], OVs have been widely accepted as a novel treatment for solid tumor in clinic. However, the durable response rate of T-VEC was only 16.3% in advanced melanoma patients [5]. How to improve the efficacy of OVs is a major challenge for clinical practice. T1012G studied in this project is a type I herpes simplex virus (HSV-1) which could be genetically engineered easily like T-VEC [7, 8]. NV1020, an oncolytic herpes virus (oHSV), has completed phase II clinical trials in the United States, which has shown safety and effectiveness in patients with colon cancer [9]. T1012G studied in this project deleted the inserted HSV-2 glycoprotein based on NV1020, which could reduce the pathogenicity of virus [7, 8].

Despite the development of novel OVs with improved efficacy and tumor selectivity, tumor resistance to OVs has been attributed to host innate immunity, namely the type I interferon (IFN-I) antiviral signaling cascade which is in the first line of defense against virus in the infected cells [10]. In response to IFN-I stimulation, STATs (STAT1, STAT2 and STAT3) are activated sequentially [11]. The activation of STAT1 and STAT2 could activate the transcription of interferon stimulated genes (ISGs) and then induce the antiviral response, while STAT3 could directly or indirectly inhibit IFN-I response and further negatively regulates IFN-I mediated antiviral response by suppressing the expression and activation of protein kinase R (PKR) [11–15]. PKR is a host antiviral kinase that effectively halts cellular proliferation and prevents production of viral proteins precluding viral replication [6]. Therefore, combining OVs with agents targeting IFN-I signaling cascade is a rational approach to improve the anti-tumor effect of OVs [16].

Preclinical studies have confirmed that β-adrenergic signaling pathway could affect the activation of STAT3 [17, 18]. Catecholamines delivered by circulating blood or released from local sympathetic nerve fibers bind to β-adrenergic receptors resulting in Gαs-mediated synthesis of cyclic 3′–5′adenosine monophosphate (cAMP). Transient flux of intracellular cAMP activates protein kinase A (PKA) to phosphorylate multiple target proteins including β-adrenergic receptor kinase (BARK). BARK activates Src kinase, resulting in activation of transcription factor, STAT3 [17]. Therefore, we hypothesized that the replication and antitumor efficacy of OVs could be enhanced by affecting STAT3 mediated immune response through β-adrenergic signaling pathway. Using T1012G as a model OVs, we tested our hypothesis in gastric cancer cell lines and engrafted mice model.

## Methods

### Cell lines, virus and reagents

AGS (RRID:CVCL_0139) human gastric cancer cells and MFC (RRID:CVCL_5J48) murine gastric cancer cells were purchased from the Cell Bank of the Chinese Academy of Sciences (Kunming, China). The human gastric cancer cell line HGC-27 (RRID:CVCL_1279) was obtained from the Type Culture Collection of the Chinese Academy of Sciences, Shanghai, China. The Vero (RRID:CVCL_0059) cell line was obtained from the American Type Culture Collection. All cell lines were cultured in DMEM medium (Gibco,Life Technologies, China) supplemented with 10% FBS (HGC-27, AGS and MFC) or 5% newborn calf serum (vero) (Gibco, Life Technologies Australia) at 37 °C and 5% CO_2_ in tissue culture incubator. The virus T1012G was obtained by single knocking γ34.5 on the basis of wild type F strain [7, 8]. All experiments were performed with mycoplasma-free cells.

### Cell viability assay

The cells were seeded in a 96-well plate at a seeding density of 2500–3000 cells/well. After 24 h, the cells were treated with propranolol (propranolol hydrochloride, P0884, Sigma-Aldrich, U.S.A) or virus T1012G. After 48 h of treatment, the liquid in the well plate was aspirated and CCK8 activity detector (Cell Counting Kit-8, Dojindo, Japan) was added into to the wells.Then the plate should be avoided the light and placed in a 37 °C incubator, 30–60 min later, the plate was placed in a microplate reader (BioTek Epoch, U.S.A) and tested at a wavelength of 450 nm.

### Synergistic effect of T1012G plus propranolol treatment against gastric cancer cells

Cells seeded in 96-well plates (2000–3000 cells per well) were studied in the experiment of T1012G plus propranolol administration. The ‘co-treatment’ group, where the cells were infected with virus at varing concentrations (0.01,0.05,0.1,1,2,5 MOI) and at the same time, treated with propranolol for a total of 2 days. For all experiments, T1012G infection was performed for 1 or 2 h at 37 °C and cells were washed with phosphate buffered saline (PBS) before and after viral infection. Efficacies of the different modes of treatment were then evaluated by determining cell survival with the cell viability assay. Combination index (CI) values were calculated using the Chou-Talalay method to quantitatively deduce synergistic, additive or antagonistic effects of virus plus propranolol [19].

### In vitro and vivo viral replication

In vitro, the effect of propranolol on viral replication was assessed by standard plaque assay. Three dosing schedules, cotreatment and pretreatment with propranolol or virus, were evaluated for the effect on viral propagation in cultured gastric cancer cells. For cotreated samples, HGC-27 cells were infected with T1012G, propranolol was added at the same time, and the cells were incubated for 24 and 48 h in HGC-27. For pretreated samples, HGC-27 cells were incubated for 12 h in the presence of propranolol and then infected with virus. As for the virus pretreatment group, the pretreatment time was the same as that in the drug pretreatment group on these cells. propranolol at low toxicity concentrations was used and the virus was stored in milk 24 or 48 h after treatment with different sequential drugs and viruses in tumor cells. After repeated freezing and thawing three times, the virus was added to the pre-paved vero cells. After 3 days of infection, the number of plaques was calculated, and the virus concentration under different treatments was obtained. In the in vivo HGC-27 animal model, tumor tissues were collected 14 days after the last intratumoral injection of the virus, and the virus titer in the tumor tissues under different treatments was detected.

### Animal studies

HGC-27 cells was engrafted into BALB/C nude mice. About 70–80% of mice developed solid tumors in 15–20 days. Mice were divided into five groups including: blank control (PBS), propranolol (5 mg/kg for consecutive 3 days) T1012G (2 × 10^5^, 5 × 10^5^ pfu/mouse was injected intratumorally at day 1, 4 respectively), and two combined treatment groups with different administration orders. The simultaneous treatment is the same as the single respective treatments. In the propranolol-pretreated combined group, propranolol was administrated in the same way as the single drug treatment and then injected intratumorally on the 4th and 7th day. The study protocol was approved by the Ethics Committee of Xiangya Hospital, Central South University (No. 2020sydw0167) and all experiments were performed in accordance with approved guidelines of Xiangya Hospital, Central South University.

### Transient transfections of siRNAs and plasmid

HGC-27 cells were plated on 6-well plates at a density of 2 × 10^5^ per well for 24 h. Cells were then transiently transfected with 50 nM STAT3 siRNA (si-1:sequences of STAT3 siRNA: sense, GAUACGACUGAGGCGCCUATT; antisense, UAGGCGCAUCAGUCGUAUCTT; si-2: sequences of STAT3 siRNA: sense, CCACUUUGGUGUUUCAUAATT; antisense, UUAUGAAACACCAAAGUGGTT) or 1000 ng STAT3 overexpressing plasmid, using Lipofectamine RNAiMAX Reagent (invitrogen, USA) or Ribo-FECT TM CP Transfection Kit (RiboBio Co., Ltd, China) according to the manufacturer’s instructions.

### Western blot analysis

Western blot analysis was performed on cell extracts of HGC-27 cell lines pretreated with 40 μM propranolol for 2 days plus virus (0.01 MOI) for 9, 20 or 48 h. Cell lysates were quantified for protein content using a bicinchoninic acid (BCA) protein assay kit (Beyotime, Jiangsu, China). Protein samples were resolved on NuPAGE 10% Bis–Tris gels or 3–8% Tris acetate gels and then transferred to 0.45-mm nitrocellulose membrane (Bio-Rad). After saturation in Tris-buffered saline supplemented with 5% BSA, the membranes were incubated with antibodies overnight at 4 °C. The blots were detected by an imaging system (Bio-Rad, USA). Antibodies specific for the following proteins were purchased from Abcam: PKR (rabbit, 32506), phospho-Tyr446-PKR (rabbit, 32036). The antibodies specific for STAT3 (rabbit, 12640), phospho-Tyr705 (rabbit, 9145), were purchased from Cell Signaling Technology. The antibody specific for GAPDH (rabbit, KM9002) was purchased from Sungene Biotech. The antibody specific for β-actin (mouse, 66009-1-Ig) was purchased from proteintech. Antibodies specific for the viral proteins used in this study are listed as follows: HSV-1 ICP4, ICP27, ICP0 (Ackermann M, Braun DK, Pereira L, Roizman B. 1984. Characterization of herpes simplex virus 1 alpha proteins 0, 4, and 27 with monoclonal antibodies. J Virol 52:108–18); US11(Roller RJ, Roizman B (1992) The herpes simplex virus 1 RNA binding protein US11 is a virion component and associates with ribosomal 60S subunits. J Virol 66:3624–3632); VP16 (McKnight JL, Kristie TM, Roizman B (1987) Binding of the virion protein mediating alpha gene induction in herpes simplex virus 1-infected cells to its cis site requires cellular proteins. Proc Natl Acad Sci USA 84:7061–7065) ICP8 (Rumbaugh Goodwin Institute for Cancer).

### IFNα/β detection

HGC-27 cells were treated with T1012G only or T1012G and/or propranolol as described above. Culture medium was collected after 9 h, 20 h, 36 h or 9 h, 20 h infection and analyzed for IFNα/β concentration by ELISA (IFNα: CUSABIO, CSB-E08636h; IFNβ: Elabscience, E-EL-H0085c).

### Statistical analysis

Data were presented as mean ± SEM. Significant differences were evaluated using one-way ANOVA or unpaired *t*-test. Differences were considered significant if the P value was less than 0.05. All statistical analyses were performed using GraphPad Prism software (GraphPad Software, Inc., version 8.0).

## Results

### Cotreatment of T1012G and propranolol exerted a synergistic killing effect in gastric cancer

**Fig.1:**
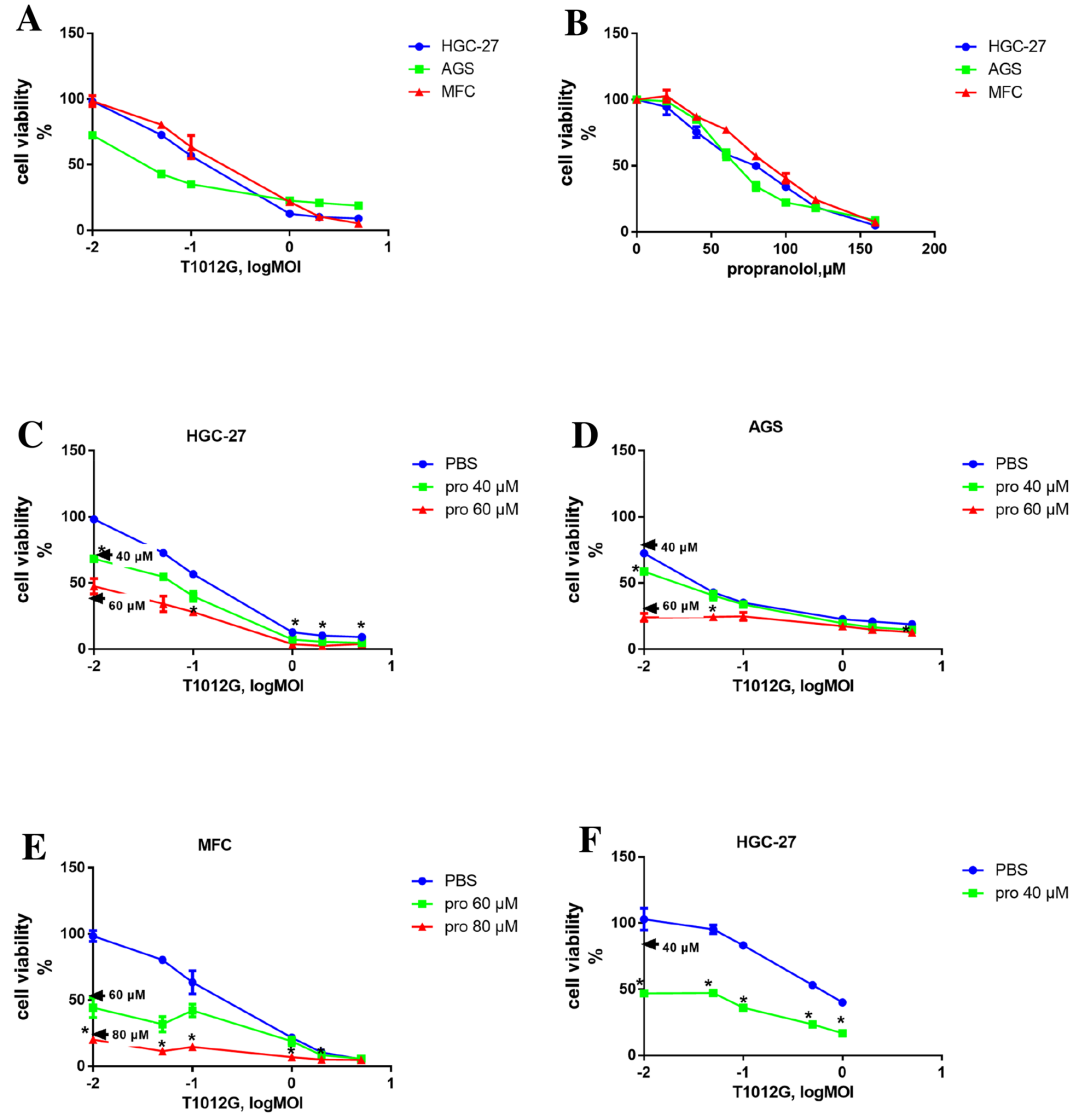
Efficacy of cell killing by oncolytic T1012G and propranolol as separate agents and combined treatment on cell proliferation in gastric cancer cell lines. **A** and **B** CCK8 assay measured cell viability after T1012G (0.01, 0.05, 0.1, 1, 2,5 MOI) and propranolol (20, 40, 60, 80, 100,120,160 µM) treatment for 48 h. **C** and **E** The survival rate from the co-treatment of T1012G (0.01, 0.05, 0.1, 1, 2, 5 MOI) and propranolol (40, 60 µM in HGC and AGS; 60, 80 µM in MFC) at 48 h. **F** HGC-27 cells were seeded on 96-well plates. After 48 h of incubation with or without propranolol (40 μmol/l), cells were infected with different dose of virus (0.01, 0.05, 0.1, 0.5, 1 MOI). The number of surviving cells in each well was determined 2 days after infection. Data are presented as mean ± SEM. Significant differences were evaluated using one-way ANOVA, and the asterisk (*) indicates a significant difference compared the same T1012G dose or propranolol alone with the combination group using Games-Howell's multiple comparisons test (*P* < 0.05). Pro, propranolol. 40/60/80 µM → indicates the cell viability corresponding to propranolol treatment alone

The IC50 of T1012G were determined to be 0.15 MOI, 0.04 MOI, and 0.21 MOI in HGC-27, AGS and MFC cell lines, respectively and the IC50 of propranolol were 70 μM, 67 μM and 86 μM in the three cell lines (Fig. 1A and B). The combined therapy of these two agents exhibited enhanced inhibition on cell viability in a concentration dependent manner (Fig. 1C–E). The synergistic effect was measured by combination index (CI) using Chou-Talalay algorithm. The lowest CI values (0.523, 0.607, and 0.657) were observed in co-treatment group 60 μM + 1MOI, 80 μM + 0.05MOI and 60 μM + 0.01MOI in HGC-27, AGS, MFC cell lines, respectively (Table 1 and Additional file 1: Table S1). In addition, pre-treated propranolol exhibited stronger synergistic effect than co-treatment model in HGC-27 (Fig. 1F). Under low dose virus infection (0.01, 0.05 and 0.1 MOI), the CI value of the pre-treatment group was significantly lower than that of the co-treatment group (0.549 vs. 1.023 *P* < 0.05, 0.624 vs. 0.944 *P* < 0.05, 0.540 vs. 0.829 *P* < 0.05) (Table 1).

**Table 1:** Combination index (CI) values for propranolol and T1012G combination for HGC-27 gastric cancer cell lines

	Propranolol (40 μM) + T1012G (0.01MOI)	
	Propranolol + T1012G	Propranolol → T1012G
CI^a^	1.023 ± 0.035	0.549 ± 0.017
Combination effect	Nearly additive ±	Synergism+++

	Propranolol (40 μM) + T1012G (0.05MOI)	
	Propranolol + T1012G	Propranolol → T1012G
CI^a^	0.944 ± 0.008	0.624 ± 0.042
Combination effect	Nearly additive ±	Synergism+++

	Propranolol (40 μM) + T1012G (0.1MOI)	
	Propranolol + T1012G	Propranolol → T1012G
CI^a^	0.829 ± 0.130	0.540 ± 0.049
Combination effect	Moderate synergism++	Synergism+++

^a^Combination index [±: nearly additive (CI 0.90–1.10); +: slight synergism (CI 0.85–0.90); ++: moderate synergism (CI 0.70–0.85); +++: synergism (CI 0.30–0.70)] (55) The CI value of combination models were measured by Chou-Talalay method where CI value quantitatively defines synergism (CI < 1), additive effect (CI = 1) and antagonism (CI > 1)

Data are presented as mean ± SEM, *P* < 0.05 (unpaired *t*-test)

### Propranolol pretreatment enhanced the propagation of T1012G in vitro

**Fig. 2:**
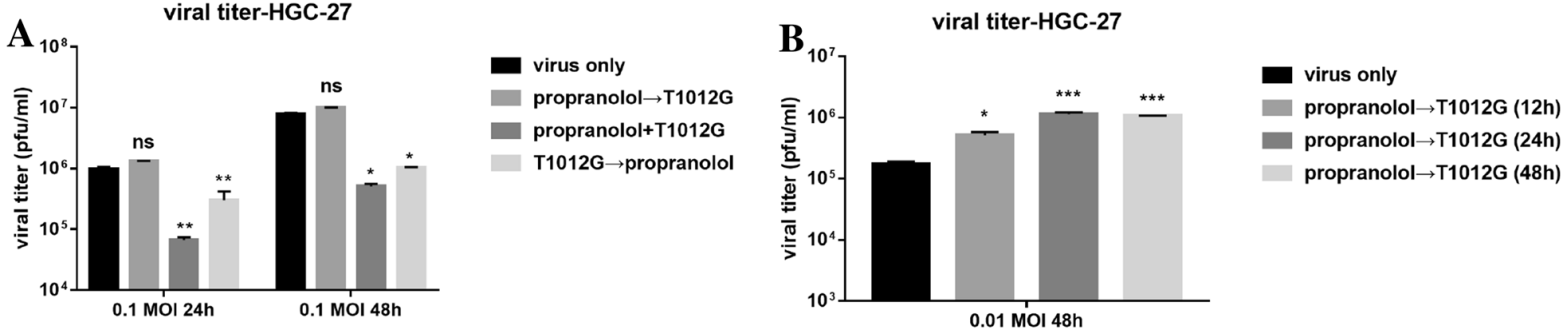
Propranolol enhanced the propagation of oncolytic herpes simplex virusT1012G in vitro.** A** Viral replication assay was applied to measure the propagation of T1012G (0.1 MOI) in HGC-27 either co-treated or pre-treated or post-treated by propranolol (40 μmol/l). The titer of T1012 was measured 24 h and 48 h after infection. **B** T1012G (0.01 MOI) was pre-treated by propranolol (40 μmol/l) in HGC-27 for 12, 24 or 48 h. The titer of virus was measured 48 h after infection. Data are presented as mean ± SEM. *P < 0.05, **P < 0.01, ***P < 0.001 vs. T1012 only (Tukey test for multiple comparisons)

Propranolol (40 μmol/l) pre-treatment for 12 h also did not affect T1012G (0.1 MOI) replication in HGC-27 cells (Fig. 2A, P > 0.05). However, when the load of T1012G was decreased to 0.01 MOI and the pre-treatment was extended to 12, 24 and 48 h, propranolol (40 μmol/l) yield a three, seven, sixfolds increase of T1012G titers respectively when compared with T1012G only group(Fig. 2B, P < 0.05, *P* < 0.001, *P* < 0.001). This data suggested that a low dose of OVs is needed when it is used in combination with propranolol. On the other hand, the co-treatment and T1012G (0.1 MOI) pre-treatment could significantly shut down T1012G replications in HGC-27 cells (Fig. 2A), which suggested the importance of the sequential administration of these two drugs.

### Propranolol pretreatment inhibited tumor growth and enhanced T1012G propagation in vivo

**Fig. 3:**
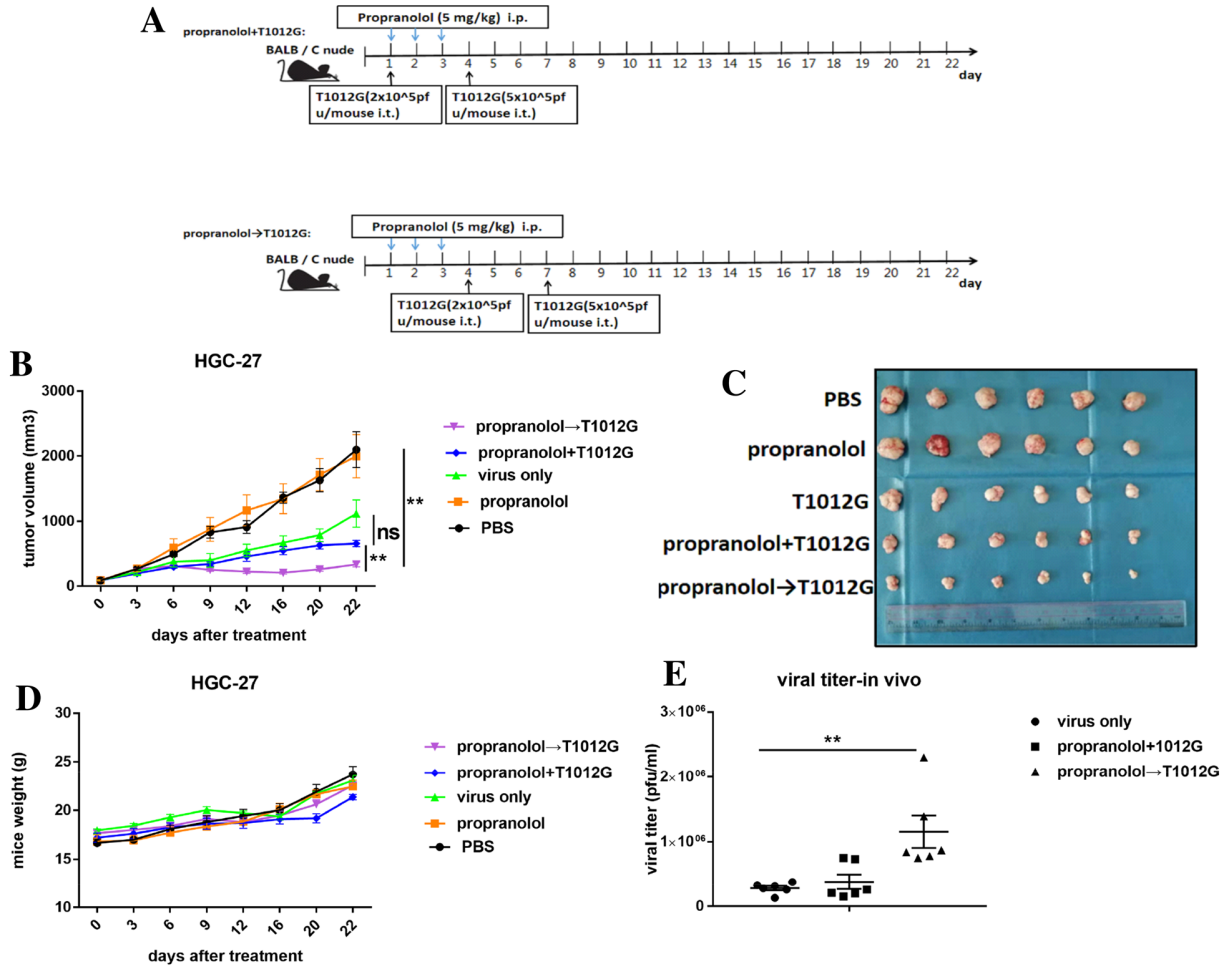
Propranolol pretreatment improved the T1012G mediated antitumor efficacy in a human gastric cancer xenograft model in nude mice. **A** Schematic plan for the administration of PBS, propranolol, T1012G, Prop + T101G, Prop → T1012G to tumor-bearing mice. **B** and **C** Tumor growth curves was plotted by average volume of six tumors for each group. **D** Body weight of mice were measured on day 0 and every three days thereafter. **E** Titers of progeny T1012G in the tumors were determined by standard plaque assays on vero cells. Results are presented as mean ± SEM. Significant differences were evaluated using one-way ANOVA. **P < 0.01 (Games-Howell’s multiple comparisons test or Dunnett’s multiple comparison test)

The synergistic effect of propranolol and T1012G was assessed in HGC-27 tumor engrafted in BALB/C nude mice. The mean tumor sizes of propranolol pre-treated mice were smaller than propranolol co-treated group (335.3 ± 36.92 mm^3^ vs. 659.3 ± 49.26 mm^3^, *P* < 0.01; Fig. 3B) and PBS group (335.3 ± 36.92 mm^3^ vs. 2100 ± 275.4 mm^3^, *P* < 0.01; Fig. 3B) on day 22 in HGC-27 tumor models. There was no statistical difference between T1012G only group and propranolol co-treated group (1118 ± 210.0 mm^3^ vs. 659.3 ± 49.26 mm^3^, *P* > 0.05; Fig. 3B). The differences in body weight of the mice were not observed among groups (Fig. 3D). The titers of T1012G were measured via standard plaque assay. Propranolol pre-treatment significantly increased T1012G replication in tumors when compared with T1012G only group (1.15 × 10^6^ ± 2.5 × 10^5^ pfu/ml vs. 2.84 × 10^5^ ± 3.5 × 10^4^ pfu/ml, *P* < 0.01; Fig. 3E), while there was no statistical difference between propranolol co-treated group and T1012G only group (3.84 × 10^5^ ± 3.5 × 10^4^ pfu/ml vs. 2.84 × 10^5^ ± 1.1 × 10^5^ pfu/ml, *P* > 0.05; Fig. 3E). Overall, these results indicated that pretreatment with propranolol can enhance the efficacy of T1012G in gastric cancer models, possibly because of the elevated T1012G titers in tumors.

### Propranolol pretreatment enhanced the expression of viral proteins and affected T1012G induced antiviral immune response

**Fig. 4:**
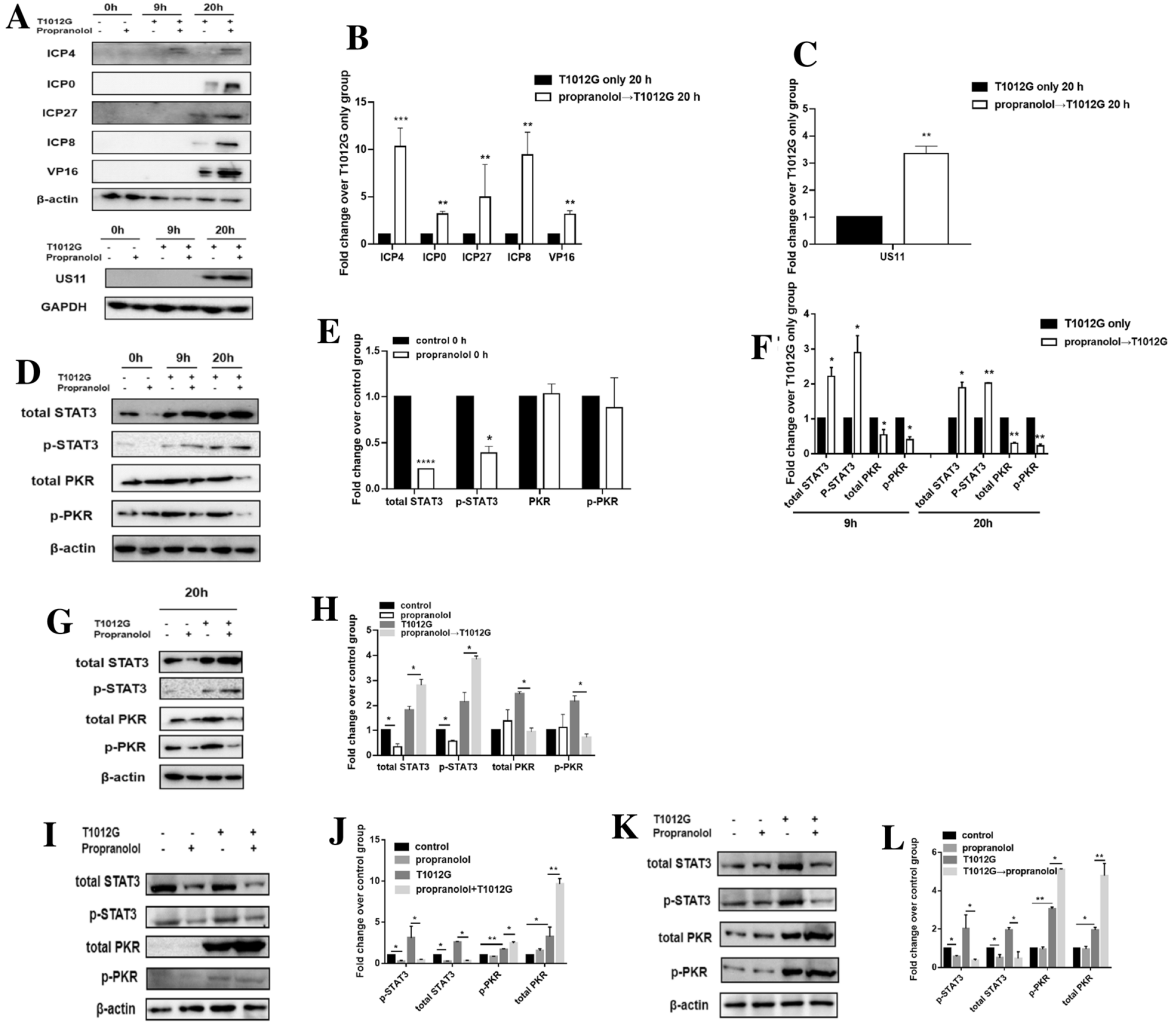
Propranolol pretreatment affected the expression of viral proteins and interferon responsive genes.** A** Western blotting of viral proteins in HGC-27 cells. After 48 h incubation with or without 40 μmol/l propranolol, cells were infected with 0.01 MOI of T1012G. Cells were harvested at indicated time points and subjected to western blotting analysis using antibodies specific to these different stages of viral proteins. The GAPDH was used as a loading control. ICP, infected-cell protein. **D** The expression and phosphorylation of interferon responsive gene was measured at 0, 9 and 20 h after T1012G treatment by western blotting. **G** Cells were pretreated with 40 μmol/l propranolol for 48 h and then infected with 0.01 MOI of virus. Cells were harvested after T1012G treatment for 20 h. **I** Cells were treated with 40 μmol/l propranolol and 0.01 MOI of virus for 20 h. Cells were harvested after cotreatment for 20 h. **K** Cells were infected with 0.01 MOI of virus for 8 h and then treated with 40 μmol/l propranolol for 12 h. Cells were harvested after T1012G treatment for 20 h. **B** and **C**, **E** and **F**, **H**, **J**, **L** Quantification of A, D, G, I, K. Data are presented as mean ± SEM, significant differences were evaluated using unpaired *t*-test or one-way ANOVA. **P < 0.01, ***P < 0.001 vs. T1012 only (unpaired *t*-test). *P < 0.05, **P < 0.01 and ****P < 0.0001 (Tukey test for multiple comparisons or Dunnett’s multiple comparisons test)

We next examined the expression of viral proteins at different stage of infection including immediate early viral proteins (ICP4, ICP0, ICP27), early viral protein (ICP8), and late viral proteins (VP16, US11). After 20 h infection, ICP4, ICP0, and ICP27 was increased 10.3-fold, 3.2-fold, and 5.0-fold compared with T1012G only group, respectively (Fig. 4A and B, *P* < 0.001, *P* < 0.01, *P* < 0.01,). The ICP8 was significantly upregulated 9.4 fold compared with T1012G only group (Fig. 4A and B, *P* < 0.01). A 3.2-fold and 3.4-fold increase of VP16 and US11 was observed between these groups (Fig. 4A–C, *P* < 0.01, *P* < 0.01). These data indicated that propranolol pre-treatment enhanced propagation of T1012 by facilitating the synthesis of vital viral proteins at different stages.

In order to detect specific mechanism, we found that the phospho-STAT3 (p-STAT3) and total STAT3 was inhibited after a 48 h treatment of propranolol (40 μM) compared with untreated group (Fig. 4D and E, *P* < 0.05, *P* < 0.0001). After 9 and 20 h infection of T1012G, p-STAT3 in the propranolol pretreatment group was significantly up-regulated 2.9 and 2.0-fold compared with T1012G only group (Fig. 4D and F, *P* < 0.05, *P* < 0.01) and total STAT3 was increased 2.3 and 1.9-fold (Fig. 4D and F, *P* < 0.05, *P* < 0.05), while the expression and activation of other IFN-I responsive genes-STAT1, STAT2 were not affected significantly (Additional file 1: Figure S1, *P* > 0.05). Meantime, phospho-PKR (p-PKR) increased 2.2-fold in T1012G only group when compared with control group (Fig. 4G and H, *P* < 0.05), suggesting that the activation of PKR may play an important role in mediating antiviral response. However, propranolol pretreatment induced a 62.46% ± 5.06%/65.94% ± 10.11% decrease of total-PKR/p-PKR compared with T1012G only group (Fig. 4G and H, *P* < 0.05) indicating a potent suppression on interferon induced antiviral response. These data suggested that propranolol pretreatment could inhibit the virus induced expression of antiviral genes PKR through enhancing the activation of STAT3. However, cotreatment or virus pretreatment could inhibit the expression of p-STAT3 and total STAT3 compared with virus only group (Fig. 4I–L, *P* < 0.05, *P* < 0.05 or *P* < 0.05, *P* < 0.05). Meantime, p-PKR and total PKR increased 1.5-fold and 3.0-fold or 1.7-fold and 2.4-fold in cotreatment or virus pretreatment group when compared with virus only group (Fig. 4I–L, *P* < 0.05, *P* < 0.01 or *P* < 0.05, *P* < 0.01).

### STAT3 altered HSV-1 propagation and cytotoxicity in gastric cancer cells by inhibiting IFN-I antiviral pathway in response to T1012G infection

**Fig. 5:**
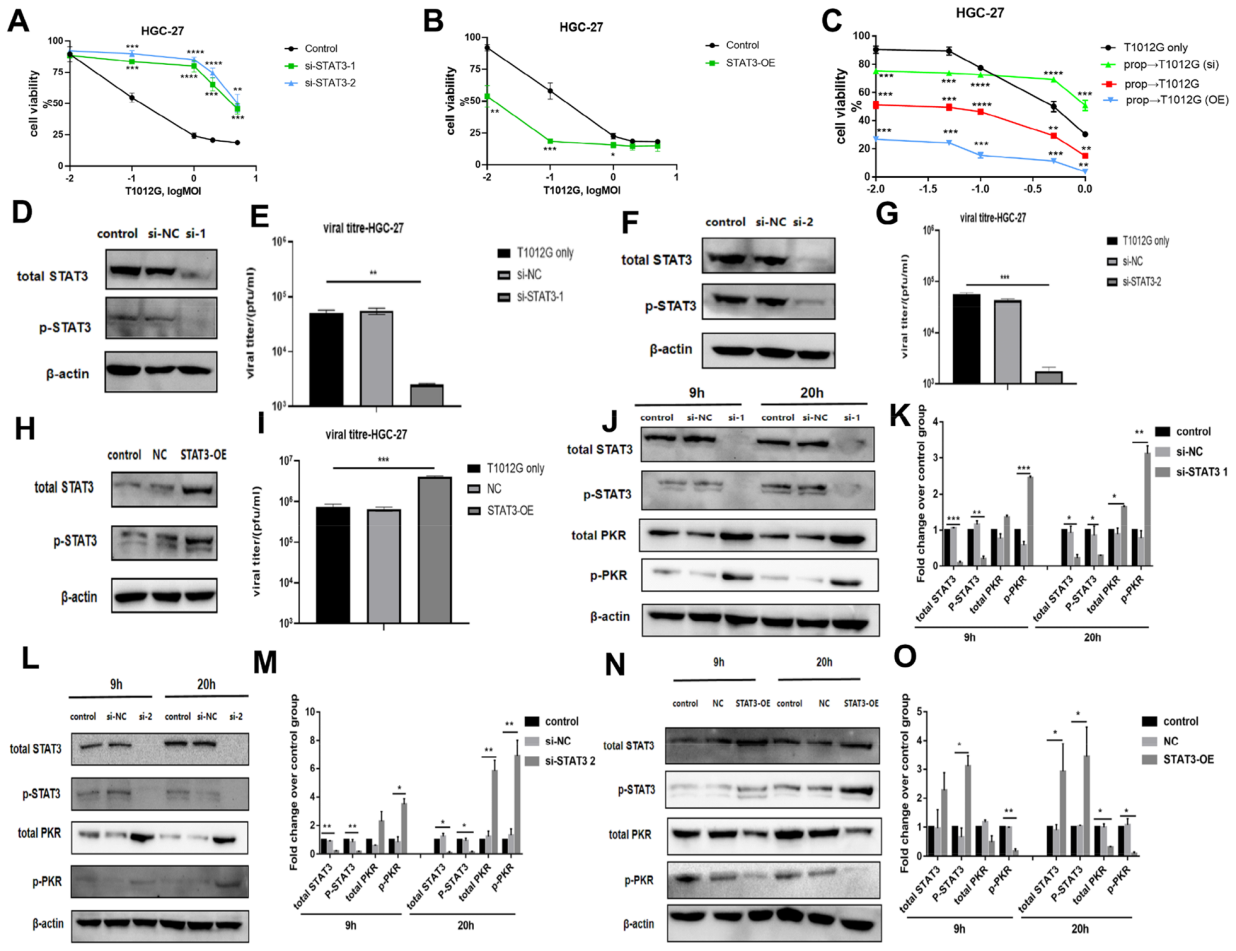
Cytotoxicity of T1012G against gastric cancer cells, virus replication and expression of IFN-I response genes after T1012G infection with altered STAT3 gene expressions. **A** and **B** Cell viability (measured by cck8) of HGC-27 gastric cancer cells transfected with siRNA or plasmid was assayed 2 days after infection of T1012G at different MOI. **C** After 48 h of incubation with or without propranolol (40 μmol/l), cells transfected with control treatment, siRNA or plasmid were infected with different dose of virus (0.01, 0.05, 0.1, 0.5, 1 MOI). The number of surviving cells in each well was determined 2 days after infection. **D**, **F** and **H** Western blot analysis of STAT3 in the transfected siRNA (si-1 and si-2) and plasmid. **E**, **G** and **I** Cells were infected (MOI 0.01) with HSV-1 (T1012G) and virus yields were determined on Vero cells after 48-h infection. **J**, **L** and **N** Expression of IFN-I responsive genes, PKR, 9 or 20 h following T1012G infection (0.01 MOI) in **J** and **L**: HGC-27 cells treating with STAT3 siRNA or vehicle and **N**: HGC-27 cells treating with plasmid-STAT3 or vehicle. **K**, **M** and **O** Quantification of J, L, N. Results are presented as mean ± SEM, significant differences were evaluated using one-way ANOVA. *P < 0.05, **P < 0.01, ***P < 0.01 and ****P < 0.0001 (Tukey test for multiple comparisons). **P < 0.01 and ***P < 0.01 (Dunnett's test for multiple comparisons). *OE* overexpression

Human HGC-27 gastric cancer cells were transfected with siRNA target to STAT3 (si-1, si-2) and si-NC (negative control). The cytotoxicity of HSV-1 against cells was measured with STAT3 knockdown by si-STAT3 (Fig. 5A) or with STAT3 overexpression (Fig. 5B). Cells with STAT3 knockdown exhibited virus cytotoxicity at an IC50 of 6.2 MOI and 5.5 MOI, when compared to control cells (IC50 of 0.18 MOI). Conversely, overexpressing cells exhibited cytotoxicity that was measured with an IC50 of 0.009 MOI compared with control cells (IC50 of 0.195 MOI). Genetic manipulation of STAT3 altered T1012G-mediated cytotoxicity of gastric cancer cells. Figure 5C showed that propranolol pretreatment enhanced the cytotoxicity of T1012G, meantime, this effect could be reversed by knocking down STAT3 or be enhanced by STAT3 overexpression.

In contrast to parental cells that expressed basal levels of STAT3, there was 94.97% ± 0.30% and 96.89% ± 0.28% reduction in viral production in HGC-27 with STAT3 knocked down (Fig. 5E and G, *P* <  0.01, *P* < 0.001). Meantime, a sixfold increase of viral titers was observed in STAT3 overexpressed HGC-27 cell lines compared with control group (Fig. 5H and I, *P* < 0.001). Collectively, this data showed that STAT3 expression positively correlates with HSV-1 replication.

Figure 5J and L showed that, n or 20 h after T1012G infection, cells with STAT3-knockdown can induce 2.4-fold, 3.5-fold or 3.1-fold, 6.9-fold increase in the expression of p-PKR (Fig. 5J–M, ***P < 0.01, *P < 0.05, **P < 0.01, **P < 0.01). Conversely, cells with STAT3-overexpression caused 82.51% ± 8.14% or 90.63% ± 5.96% decrease in the expression of p-PKR (Fig. 5N and O, **P < 0.01, *P < 0.05), while total PKR in cells transfected with siRNA or plasmid was not altered (Fig. 5J–O, *P* > 0.05). These results suggested that the increase of viral replication in HGC-27 is mediated by STAT3-suppressed IFN-I signaling cascade.

### Propranolol pretreatment counteracted IFN-α/β-mediated inhibition of viral propagation

**Fig. 6:**
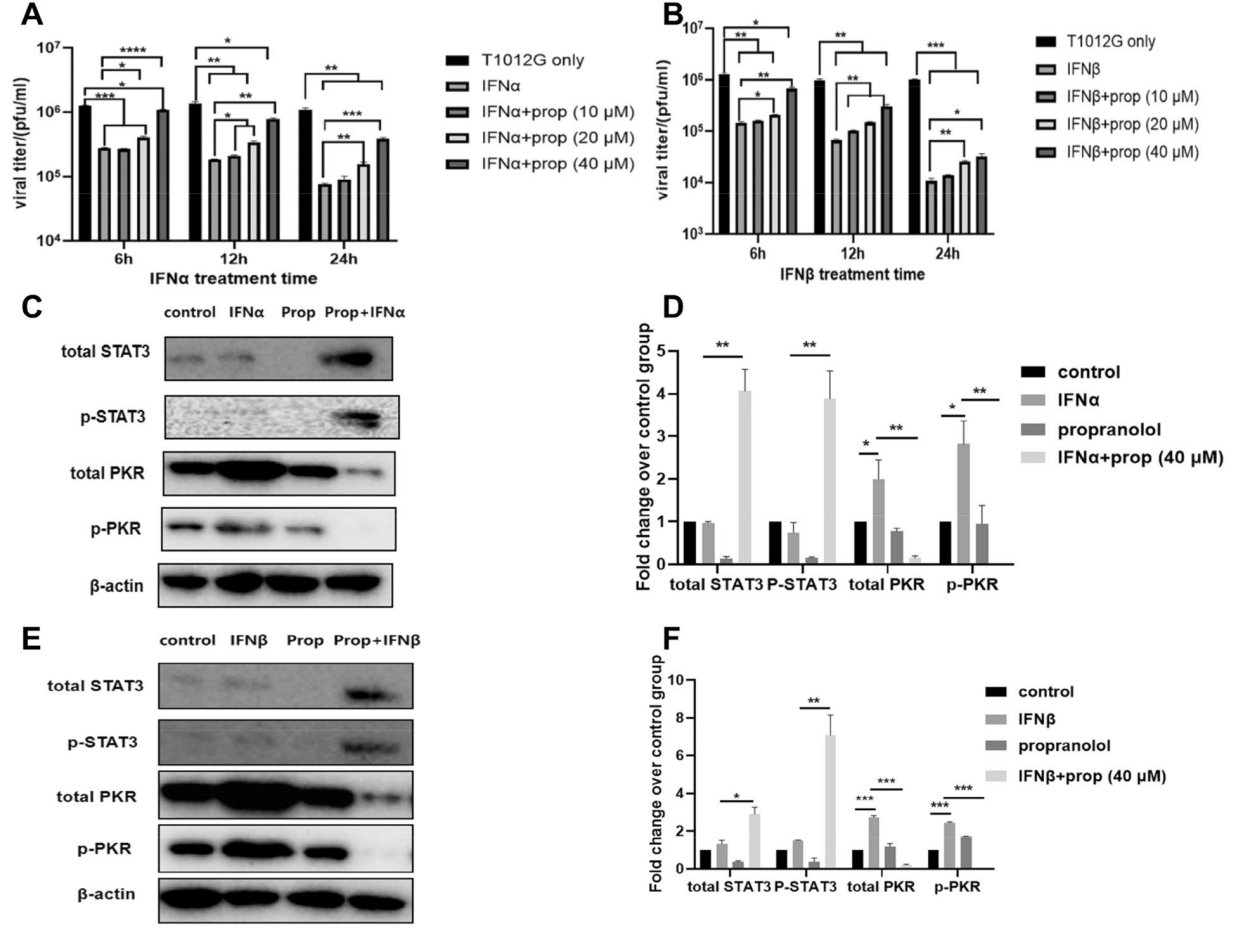
Propranolol treatment inhibited the IFN-I-mediated induction of antiviral genes and counteracted IFN-mediated inhibition of viral propagation in HGC-27 human gastric cancer cells. **A** and **B** Dose effects of propranolol were evaluated on the IFN-induced inhibition of T1012G production in HGC-27 cells. Cells were treated with 300 ng/well of human recombinant IFN-α/β and different concentrations (0, 10, 20, or 40 μmol/l) of propranolol for 48 h and infected withT1012G (0.01 MOI). Two days after infection, the cells and medium were harvested, and yields of virus were determined were determined on Vero cells. **C** and **E** HGC-27 cells were incubated with 40 μmol/l propranolol (for 48 h) and/or 300 ng/well human recombinant IFN-α/β (for 6 h) and then harvested for the assessment of protein expression of STAT3, p-STAT3, PKR, and p-PKR by western blot analysis. **D** and **F** Quantification of C, E. Results are presented as mean ± SEM, significant differences were evaluated using one-way ANOVA. *P < 0.05, **P < 0.01, ***P < 0.001(Dunnett’s test for multiple comparisons)

IFN-α/β treatment inhibited the replication of T1012G by > 70% in HGC-27 cells at 6 h (Fig. 6A and B, *** *P* < 0.01). With the prolongation of interferon treatment time, the degree of virus replication inhibition was greater than 6 h treatment (~ 90% reduction). Propranolol treatment counteracted IFN-α/β in a dose-dependent manner in cells infected with T1012G (Fig. 6A and B). The 40 μM propranolol treatment cotreated with IFN-α/β increased the viral yields (compared with IFNα: 3.9-fold, 4.2-fold, 5.1-fold; IFNβ: 4.6-fold, 4.6-fold, 3.0-fold) (Fig. 6A and B). The expression of PKR or p-PKR was significantly upregulated 2.0-fold/2.7-fold or 2.8-fold/2.5-fold in response to IFN-α/β (Fig. 6C–F, *P < 0.05, ***P < 0.001, *P < 0.05, ***P < 0.001), and the IFN-mediated upregulation was significantly attenuated (91.57% ± 4.40%/92.57% ± 1.77% or 99.63% ± 0.16%/99.34% ± 0.13% decrease) by propranolol treatment (Fig. 6C–F, **P < 0.01, ***P < 0.001, **P < 0.01, ***P < 0.001), while the cotreatment with propranolol and IFN-α/β induced 4.2-fold/2.2-fold or 5.5-fold/4.7-fold increase in total STAT3 or p-STA3 compared to IFN-α/β treatment (Fig. 6C–F, **P < 0.01,*P < 0.05, **P < 0.01, **P < 0.01). These results indicated that propranolol pretreatment could counteract the actions of IFNs and rescue viral yields by preventing IFN-mediated upregulation of PKR.

### Virus infection enhanced the secretion of IFNα, and propranolol pretreatment could further enhance its secretion

**Fig. 7:**
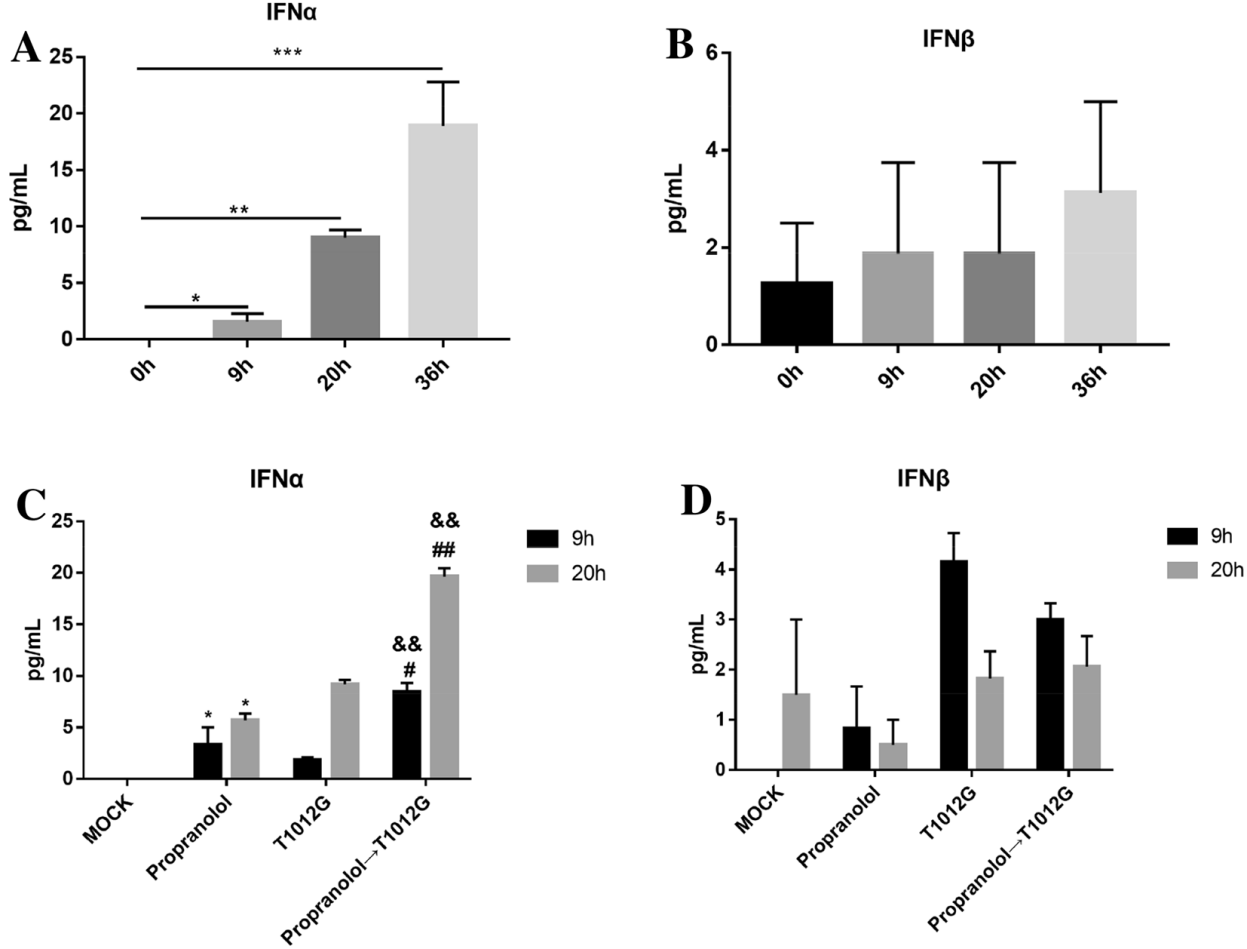
Virus infection promoted the secretion of IFNα and combined treatment could further enhance its secretion. **A** and **B** Cells were infected with T1012G (0.01 MOI). Culture medium was collected and analyzed for IFNα or IFNβconcentration by ELISA after 9 h, 20 h and 36 h infection. **C** and **D** Cells were pretreated with 40 μmol/l propranolol for 48 h and then infected with 0.01 MOI of virus. Culture medium were harvested after T1012G treatment for 9 h and 20 h. Results are presented as mean ± SEM, significant differences were evaluated using one-way ANOVA. *P < 0.05, **P < 0.01, ***P < 0.001; ^#^*P* < 0.05, ^##^*P* < 0.01 vs. propranolol group; ^&&^*P* < 0.01 vs. T1012G group (Dunnett’s test for multiple comparisons)

With the prolongation of virus infected time, the secretion of IFNα increased continuously (compared with oh: 9 h: *P < 0.05; 20 h: **P < 0.01; 36 h: ***P < 0.001; Fig. 7A), while there was no obvious effect on the secretion of IFNβ (*P* > 0.05; Fig. 7B). Meantime, we also found that propranolol treatment could slightly enhance IFNα secretion compared with untreated group, and propranolol pretreatment could further enhance IFNα secretion compared with single treatment group after 9 h or 20 h virus infection (9 h: compared with propranolol group: #*P* < 0.05; compared with T1012G group: ^&&^*P* < 0.01; 20 h: compared with propranolol group: ^##^*P* < 0.01; compared with T1012G group: ^&&^*P* < 0.01;Fig. 7C), while there was no significant effect on the secretion of IFNβ (*P* > 0.05; Fig. 7D). These results indicated that propranolol pretreatment may further enhance virus replication by enhancing the secretion of IFNα and then promoting the activation of its downstream product-STAT3.

## Discussion

This study elucidated that β-blocker pretreatment improved the antitumor efficacy of an oncolytic virus, T1012G, by regulating STAT3-PKR dependent antiviral response in gastric cancer cells in response to viral infection and type I IFNs. This data firstly demonstrated that β-adrenergic receptor inhibition could provide optimal survival conditions for OVs, presumably by enhancing intracellular viral replication.

Activation of β-adrenergic receptor signaling by catecholamines lead to the activation of a series of kinases, then cause the activation of the transcription factors, STAT3 sequentially [17, 18]. β-blockers were believed to suppress the signaling cascade and inhibit STAT3 expression and activation, which was seen in this study (Fig. 4G). Interestingly, the following virus infection abrogates the inhibitory effects of β-blocker on STAT3, causing a drastically increased expression of STAT3/p-STAT3 along with a decrease of PKR/p-PKR. Apparently, β-blocker pretreatment caused antiviral response was reversed to a pro-viral infection response after the administration of virus. T1012G infection could promote type I IFN secretion (Fig. 7A). Using IFNα/β to mimic the antiviral response induced by virus infection, we found similar changes of STAT3 and PKR (Fig. 6C–F) which indicated that the abrogation of the antiviral response by β-blocker could be caused by virus induced secretion of type I IFN. Similar result was seen in Otsuki and Okemoto’s report who found that using valproic acid (VPA) during OV infection did not improve the replication and spread of the virus, but pre-treatment of VPA was sufficient to increase OV productivity in glioma cells [20, 21]. These data indicated that the timing of drug treatment was critical for OV replication in tumor cells. In addition, consistent with our study they also found that the enhancement effect of VPA pretreatment on viral replication was mainly through increased STAT3 activation and thereby decreased ISGs expression (PKR, etc.).

Low response rate is the major limitation of practicing OVs in clinic. Current solution is focusing on combining the virus with the agents that could suppress the host innate immune response. Fulci et al. found that suppressing host innate immunity with cyclophosphamide could significantly enhance the replication of HSV-1 based OV in brain tumors, thus enhancing its antitumor efficacy [22]. In another study, sunitinib (Sutent), a potent inhibitor of both antiviral enzyme RNase L and PKR, could suppress the antiviral innate immunity and then enhance oncolytic virotherapy [23]. In this study, we demonstrated that β-blocker pretreatment could enhance virotherapy through enhancing viral replication. T1012G is currently used as a tool drug to explore the anti-tumor effect of oncolytic HSV-1 [7, 8]. It’s rational to believe that other OVs (including T-VEC, HF10, G207) would likely to benefit from β-blocker pretreatment. Meantime, the effect of β-blocker in combination with OVs in other cancers needs further exploration. According to the conversion formula between experimental animal dose and human clinical dose, the dose of propranolol used in this study (38 mg/d) is lower than the actual clinical dose (90 mg/d). Pretreatment with low dose propranolol and then intratumoral injected with low dose oncolytic virus T1012G could significantly enhance the anti-tumor effect of virus (Fig. 3A–C). The combination therapy could not only effectively reduce the dose of virus used, but also significantly enhance the anti-tumor effect of virus. Clinical trials are also needed to verify the synergistic effect of the combined treatment of β-blocker with OVs in gastric cancer patients as an adjuvant regimen.

## Conclusions

In summary, this data implied that the combination of β-blocker with OV could exert synergistic antitumor efficacy in vitro and in vivo. The study highlights for the first time that β-blocker can improve the efficacy of tumor virotherapy in gastric cancer and preloaded β-blocker could enhance viral replication through promoting the secretion of IFNα and further upregulating the p-STAT3 and then inhibiting the induction of the IFN-responsive antiviral gene-PKR, even in the presence of type I IFNs.

## Supplementary Information

**Additional file 1: Table S1. **The CI value of combination models were measured by Chou-Talalay method [19]. **Figure S1.** Propranolol pretreatment possessed no effect on the expression and activation of interferon responsive genes-STAT1, STAT2. (A, D) The expression and phosphorylation of interferon responsive genes (STAT1 and STAT2) were measured at 0, 9 and 20 hours after T1012G treatment by western blotting. (B–C, E–F) Quantification of A, D. Data are presented as mean±SEM.

## Data Availability

The data that supports the findings of this study are available from the corresponding author upon reasonable request.
