# Supplementary material for: β-adrenergic receptor inhibition enhances oncolytic herpes virus propagation through STAT3 activation in gastric cancer
Source: Cell Biosci. 2021 Sep 20;11:174. doi: 10.1186/s13578-021-00687-1 (PMC8454049; doi:10.1186/s13578-021-00687-1)
Supplement: Supplementary file 1 — Additional file 1: Table S1. The CI value of combination models were measured by Chou-Talalay method [19]. Figure S1. Propranolol pretreatment possessed no effect on the expression and activation of interferon responsive genes-STAT1, STAT2. (A, D) The expression and phosphorylation of interferon responsive genes (STAT1 and STAT2) were measured at 0, 9 and 20 hours after T1012G treatment by western blotting. (B–C, E–F) Quantification of A, D. Data are presented as mean±SEM. [file 13578_2021_687_MOESM1_ESM.docx]

**Supplementary Information**

**Table S1** CI values for propranolol and T1012G cotreatment for AGS and MFC gastric cancer cell lines

| **MFC** | | **AGS** | |
| --- | --- | --- | --- |
| **Combination dose** | **CI^1^** | **Combination dose** | **CI^1^** |
| 60 μM+0.01 MOI | **0.712 ± 0.055** | 40 μM+0.01 MOI | **1.091 ± 0.171** |
| 60 μM+0.05 MOI | **0.643 ± 0.024** | 40 μM+0.05 MOI | **0.796 ± 0.103** |
| 60 μM+0.1 MOI | **0.843 ± 0.044** | 40 μM+0.1 MOI | **0.726 ± 0.057** |
| 60 μM+1 MOI | **1.365 ± 0.069** | 40 μM+1 MOI | **0.707 ± 0.050** |
| 60 μM+2 MOI | **1.378 ± 0.054** | 40 μM+2 MOI | **0.747 ± 0.162** |
| 60 μM+5 MOI | **2.385 ± 0.360** | 40 μM+5 MOI | **1.010 ± 0.280** |
| 80 μM+0.01 MOI | **0.692 ± 0.032** | 60 μM+0.01 MOI | **0.657 ± 0.039** |
| 80 μM+0.05 MOI | **0.607 ± 0.075** | 60 μM+0.05 MOI | **0.703 ± 0.037** |
| 80 μM+0.1 MOI | **0.694 ± 0.002** | 60 μM+0.1 MOI | **0.769 ± 0.064** |
| 80 μM+1 MOI | **0.782 ± 0.167** | 60 μM+1 MOI | **0.919 ± 0.005** |
| 80 μM+2 MOI | **0.956 ± 0.278** | 60 μM+2 MOI | **0.918 ± 0.099** |
| 80 μM+5 MOI | **1.524 ± 0.484** | 60 μM+5 MOI | **1.187 ± 0.091** |

The CI value of combination models were measured by Chou-Talalay method (19).


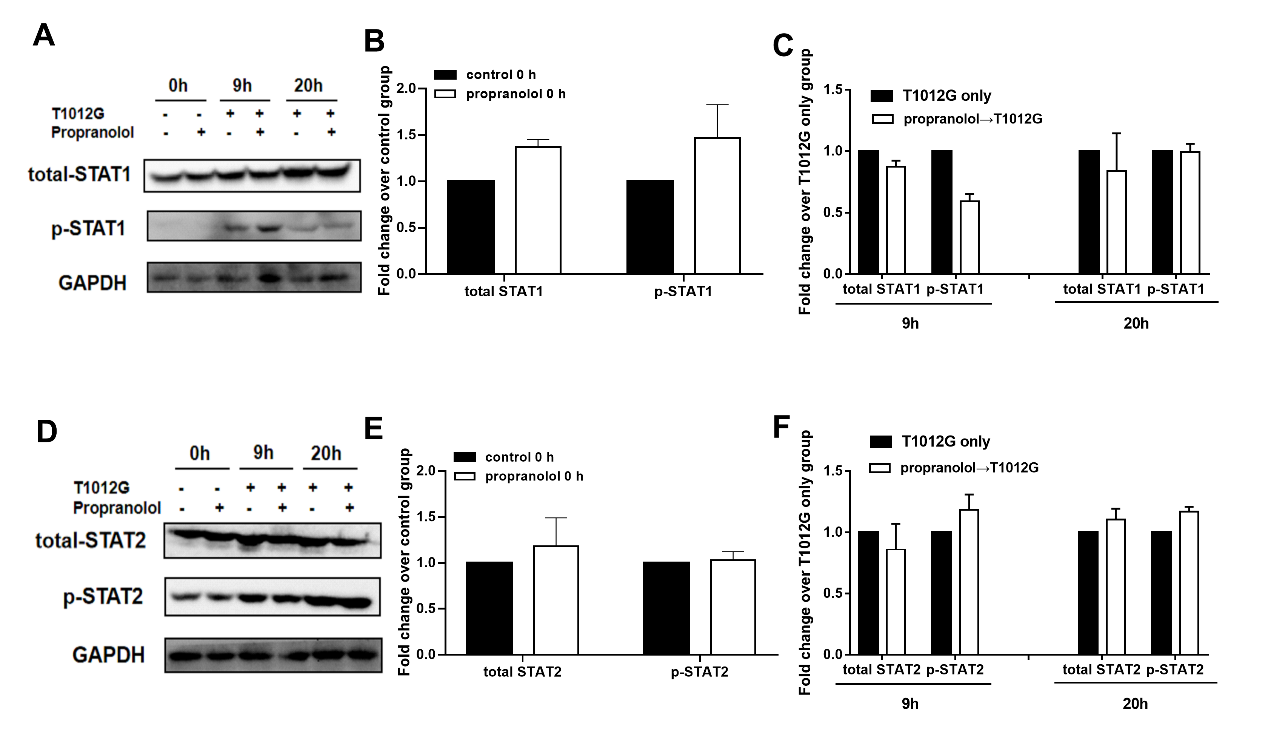


**Supplementary Figure S1.** Propranolol pretreatment possessed no effect on the expression and activation of interferon responsive genes-STAT1, STAT2. **(A, D)** The expression and phosphorylation of interferon responsive genes (STAT1 and STAT2 ) were measured at 0, 9 and 20 hours after T1012G treatment by western blotting. **(B-C, E-F)** Quantification of A, D. Data are presented as mean±SEM.
